# The Latest Hospital History

**Published:** 1913-11-29

**Authors:** 


### The Latest Hospital History.

ROTUNDA HOSPITAL, DUBLIN.

At a recent meeting of the board of governors of the Rotunda Hospital, Dublin, the Master presented to the board, in the name of Dr. T. Percy C. Kirkpatrick and himself, "The Book of the Rotunda Hospital," when it was proposed by Sir William Watson, seconded by Sir William Goulding, and resolved : "That the best thanks of the governors be given to Dr. Kirkpatrick and Dr. Jellett for the very able, interesting, and useful history which they have written of the hospital." It was proposed by Dr. Tweedy, seconded by Dr. Purefoy, and resolved : "That Dr. Percy Kirkpatrick be proposed to fill the office of governor in recognition of his eminent service to the hospital in preparing 'The Book of the Rotunda Hospital.'" The book forms the first comprehensive history of a large size which has been written of the well-known Rotunda Hospital. It deals with its subject from a very broad standpoint, and will be appreciated not merely by medical men, but by every reader who is interested in the history of Dublin during the Georgian period and afterwards. The volume is demy quarto, 222 pages, with nearly fifty full-page plates. The profits of the sale of the book will be given to the funds of the hospital.

